# Gene regulation of *Sclerotinia sclerotiorum* during infection of *Glycine max*: on the road to pathogenesis

**DOI:** 10.1186/s12864-019-5517-4

**Published:** 2019-02-26

**Authors:** Nathaniel M. Westrick, Ashish Ranjan, Sachin Jain, Craig R. Grau, Damon L. Smith, Mehdi Kabbage

**Affiliations:** Department of Plant Pathology, University of Wisconsin-Madison, Madison, WI USA

**Keywords:** *Sclerotinia sclerotiorum*, *Glycine max*, Transcriptomics, White Mold, Sclerotinia stem rot, Effectors, Hydrolytic enzymes, Resistance, Oxalic acid, Reactive oxygen species

## Abstract

**Background:**

*Sclerotinia sclerotiorum* is a broad-host range necrotrophic pathogen which is the causative agent of Sclerotinia stem rot (SSR), and a major disease of soybean (*Glycine max*). A time course transcriptomic analysis was performed in both compatible and incompatible soybean lines to identify pathogenicity and developmental factors utilized by *S. sclerotiorum* to achieve pathogenic success.

**Results:**

A comparison of genes expressed during early infection identified the potential importance of toxin efflux and nitrogen metabolism during the early stages of disease establishment. The later stages of infection were characterized by an apparent shift to survival structure formation. Analysis of genes highly upregulated *in-planta* revealed a temporal regulation of hydrolytic and detoxification enzymes, putative secreted effectors, and secondary metabolite synthesis genes. Redox regulation also appears to play a key role during the course of infection, as suggested by the high expression of genes involved in reactive oxygen species production and scavenging. Finally, distinct differences in early gene expression were noted based on the comparison of *S. sclerotiorum* infection of resistant and susceptible soybean lines.

**Conclusions:**

Although many potential virulence factors have been noted in the *S. sclerotiorum* pathosystem, this study serves to highlight soybean specific processes most likely to be critical in successful infection. Functional studies of genes identified in this work are needed to confirm their importance to disease development, and may constitute valuable targets of RNAi approaches to improve resistance to SSR.

**Electronic supplementary material:**

The online version of this article (10.1186/s12864-019-5517-4) contains supplementary material, which is available to authorized users.

## Background

*Sclerotinia sclerotiorum*, the causative agent of Sclerotinia stem rot (SSR) or white mold, is a broad host range necrotrophic fungus that infects soybean (*Glycine max*) as well as more than 400 other species of plants worldwide [1]. With conducive environmental conditions SSR can be a devastating disease, and from 2010 to 2014 caused an estimated yield loss in the northern United States and Ontario of over 100 million bushels [2]. Along with yield losses, SSR affects seed quality by reducing protein and oil contents [3].

The primary source of inoculum for *S. sclerotiorum* during seasonal crop infection cycles are sclerotia [4, 5]. Sclerotia are melanized hyphal aggregates that serve as a winter survival structure for the pathogen. These structures can undergo either myceliogenic germination, through the production of vegetative hyphae, or carpogenic germination, through the production of apothecia, the latter of which accounts for most soybean infections [6]. In the case of carpogenic germination, apothecia in the soil release millions of airborne ascospores which initially colonize the petals of soybean blossoms, before travelling through green tissue to the main stem of the plant. This journey through distinct regions of the host is important to note as the metabolic, virulence, and defense requirements of *S. sclerotiorum* may shift in response to the challenges associated with colonizing specific tissue types, such as differing levels of glucose [7], pH [8], and oxidative stress [9].

While several factors implicated in *S. sclerotiorum* pathogenicity have been characterized (Oxalic acid [10–14], secreted effectors [15–18], cell wall degrading (CWD) enzymes [19–21]), bioinformatic approaches have identified additional putative secreted proteins in *S. sclerotiorum* that may serve as virulence factors. Guyon et al [22] identified 78 effector candidates through the analysis of protein domains and motifs, signatures of positive selection, recent gene duplication, and sequences unique to *S. sclerotiorum.* Of these candidates, a subset were analyzed during penetration and colonization of *Arabidopsis thaliana*, and showed distinct patterns of expression, likely correlating with their diverse roles in infection. A comparative analysis of the genomes of *S. sclerotiorum* and its close relative *Botrytis cinerea* provided insight into their putative secretomes, but also highlighted many of the numerous hydrolytic, detoxifying, and secondary metabolite synthesizing enzymes that *S. sclerotiorum* may use during infection [23].

Although these studies have borne fruit through the identification and characterization of a number of potential virulence determinants [15, 18], *in planta* transcriptomic studies of *S. sclerotiorum* are pivotal to understanding the fungal pathogenicity determinants most important for a particular host and stage of infection. Previous studies attempting this in *Brassica napus* [24] and *Phaseolus vulgaris* [25] provide useful insight into the regulation of these determinants. The expression of a gene (*Ssoah1)* coding for an oxaloacetate acetylhydrolase, an enzyme essential in oxalic acid production, and likely the most well-studied virulence factor of *S. sclerotiorum,* was found to be similarly upregulated over the course of infection in both studies. Conversely, the expression and profiles of pectinolytic cell wall degrading enzymes (CWDEs) which are critical in fungal necrotrophy, such as polygalacturonases (PGs) [19], were differentially regulated between these two hosts. As a broad host range pathogen, *S. sclerotiorum* is capable of adapting to different hosts [26], and the characterization of host specific factors in any given crop system is essential to improve and exploit mechanisms of resistance.

Although integrated management strategies for the control of SSR in soybean are common, a historical limitation to disease control has been a lack of cultivars showing strong physiological resistance [27]. This is complicated by a polygenic resistance composed of both physiological mechanisms of resistance and structural disease avoidance traits (i.e. height, lodging, canopy closure, etc.) which might prove inconsistent with variable environmental and field conditions [28]. Recently, attempts to classify cultivars demonstrating physiological resistance using greenhouse inoculations [27] and a panel of *S. sclerotiorum* isolates for improved SSR screening [29] have identified selections showing promising resistance. In this study, we capitalize on this selection work to compare transcriptional differences between a successful/compatible *S. sclerotiorum* infection in a susceptible soybean line and one encountering localized resistance after the onset of infection.

To observe the changes in gene expression of *S. sclerotiorum* over the course of plant colonization, this study has three primary objectives: (1) identify differentially expressed genes over the course of infection in a susceptible soybean line; (2) compare gene expression in *S. sclerotiorum in planta* and under culture conditions to identify factors important in the *S. sclerotiorum*-soybean pathosystem; and (3) evaluate the differences in gene regulation during SSR infection on susceptible and resistant soybean lines.

## Results

### Disease development in susceptible and resistant soybean lines

**Fig. 1:**
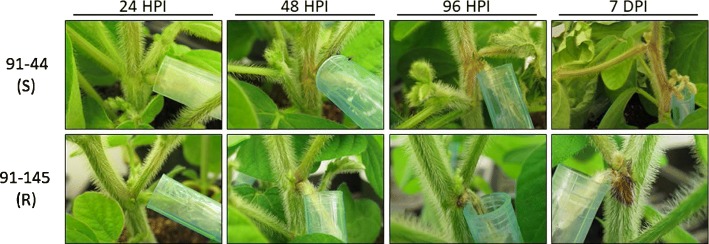
Disease symptoms observed following petiole inoculation with an agar plug containing actively growing mycelia of S. sclerotiorum at 24, 48, 96 h post-inoculation (hpi) and 7 days post inoculation (dpi). **a** Susceptible (S) line. **b** Resistant (R) line. At 7 dpi in the R line, red coloration at point of inoculation (red node) is prominently visible. The red stem phenotype developed in 90% of the R plants tested (9 out of 10), while none of the S plants showed this phenotype

*S. sclerotiorum* pathogenic development was monitored in two recombinant inbred lines (RIL) of soybean showing contrasting levels of susceptibility. The resistant 91–145 (R) and the susceptible 91–44 (S) were the result of crossing between W04–1002 (P1), a SSR resistant parental line, and LN89–5717 (PI 5745542), a parental line susceptible to SSR but demonstrating other desirable traits [27]. Plants were inoculated using the cut petiole method and infection progression was monitored for seven days. SSR symptoms, observed as typical brown lesions surrounding the point of infection, began to appear 48 h post-inoculation (hpi). At 96 hpi lesions were visible on the main stem of the S line, while disease progress had slowed substantially and restricted lesions with a red coloration were observed on the node of the R line (Fig. 1). By day 7 the susceptible soybeans showed extensive lesions along the main stem and began to wilt, whereas the resistant line had largely resisted fungal colonization into the main stem, accompanied by a more prominent red coloration at the infected node of the main stem. The red stem phenotype developed in 90% of the R plants tested (9 out of 10), while none of the S plants showed this phenotype. The red stem appears to be indicative of a specific resistance response by the R line to inhibit *S. sclerotiorum* pathogenesis and is correlated with the production of antimicrobial compounds affecting fungal growth [30]*.*

### RNA Seq analysis of *S. sclerotiorum* during infection

Global gene expression changes in *S. sclerotiorum* were determined following inoculations of the S and R lines. We sequenced the mRNA of twenty-four samples, consisting of three independent biological replicates of culture controls and infected soybean tissue at 24, 48, and 96 hpi. A total of 64.9–89.7 million raw reads were generated in each time point of the study, with 3.5 to 27% mapping to the genome of *S. sclerotiorum* and 68.9 to 92.8% mapping to the genome of *Glycine max*. On average, 96.3% of the total sequenced reads from the culture controls mapped uniquely to *S. sclerotiorum* strain 1980 genome. All reads that failed to map to either the host or pathogen genome were excluded from the analysis.

Of the reads sequenced from the S line, approximately 4.4, 4.0, and 27% mapped to the *S. sclerotiorum* genome at 24, 48 and 96 hpi, respectively. In the R line, approximately 3.5, 3.8, and 7.8% of the reads mapped to the *S. sclerotiorum* genome at 24, 48 and 96 hpi, respectively (Table 1). At 96 hpi the percentage of reads mapping to the fungal genome in the S line (27%) was significantly higher than those in the R line (8%), in accordance with the extent of tissue colonization on the stems of both lines following *S. sclerotiorum* challenge.

**Table 1:** Summary of the sequencing metrics of the RNA-seq

	Time points (hours)	Total reads	Mapping to *S. sclerotiorum*	Mapping to *Glycine max*
	Control	48,381,862	46,655,332 (96.3%)	0 (0.0%)
Susceptible line (Sus)	24 hpi	81,259,978	3,607,921 (4.4%)	74,457,988 (91.6%)
	48 hpi	74,344,340	2,941,195 (4.0%)	68,265,965 (91.8%)
	96 hpi	69,266,099	18,712,374 (27.0%)	47,765,044 (68.9%)
Resistant line (Res)	24 hpi	64,923,300	2,286,347 (3.5%)	60,261,793 (92.8%)
	48 hpi	67,520,185	2,513,457 (3.8%)	62,099,244 (91.9%)
	96 hpi	72,951,207	5 691,551 (7.8%)	64,399,890 (88.2%)

### Validation of RNA-Seq data using real-time quantitative PCR (RTq-PCR)

**Fig. 2:**
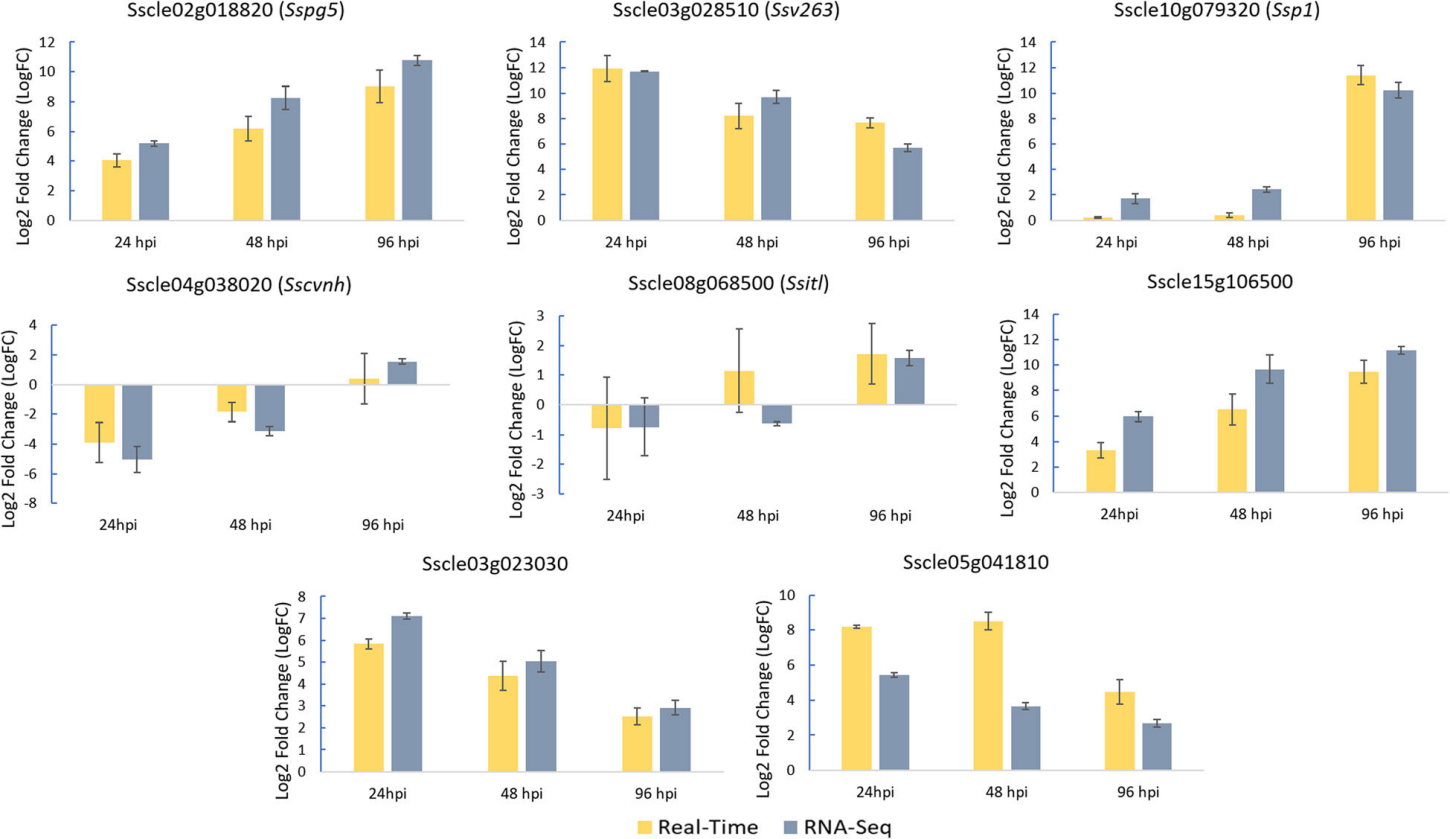
Real-time quantitative PCR (RT-qPCR) validation of RNA Sequencing (RNA-Seq) data in the S line infection. Log2 Fold Change (LogFC) values were generated for RT-qPCR samples by comparing the expression of genes at each timepoint of infection vs. the culture control using the 2 − ΔΔCt method. LogFC values were generated for RNA-Seq samples by comparing the average RPKM values of genes at each timepoint of infection vs. the culture control. Data are presented as means ± standard error (SE) from three independent replicates

To validate the accuracy of the RNA-Seq data, five previously characterized and three uncharacterized genes showing distinct expression patterns during infection of the S line were quantified using a SYBR Green based RT-qPCR. Primers were either designed or culled from the literature (Additional file 1: Table S1). The five previously characterized genes were *Sspg5* (Sscle02g018820), *Ssv263* (Sscle03g028510), *Ssp1* (Sscle10g079320), *Sscvnh* (Sscle04g038020), and *Ssitl* (Sscle08g068500). The three uncharacterized genes were a putative cytochrome p450 monooxygenase (Sscle15g106500), a putative laccase (Sscle03g023030), and a putative serine protease (Sscle05g041810). The expression patterns for each gene, as compared to culture, were similar between RNA-Seq and RT-qPCR, with *Ssitl* and Sscle05g041810 showing the greatest variance, but with similar trends between the two methods (Fig. 2). The expression level of *Ssoah1* (Sscle10g075560), which was not differentially regulated at any timepoint of infection, was also consistent between the two methods (data not shown). Thus, these results indicate a close correlation between our RT-qPCR and RNAseq data.

### *S. sclerotiorum* gene regulation over the course of a compatible interact

**Fig. 3:**
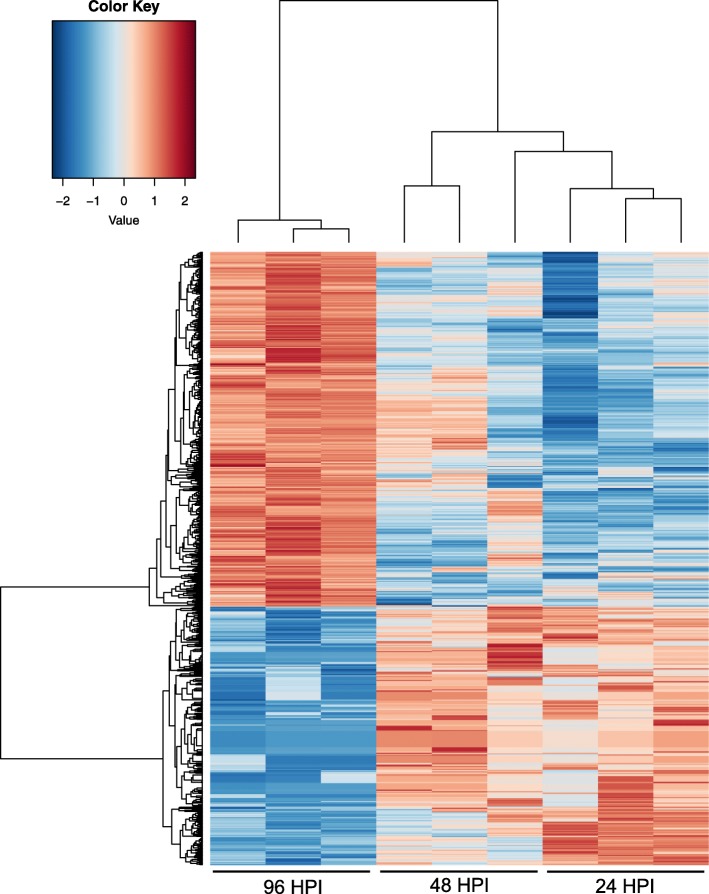
Summary heat map showing all differentially expressed genes (DEGs) in the comparison of the 96 hpi timepoint to the average of the 24 and 48 hpi timepoints. Columns represent replicates of each timepoint and rows represent individual genes. The tree above the heatmap demonstrates the hierarchical clustering of the samples. Red is used to represent genes which were up-regulated, and blue is used to represent genes which were down-regulated

To examine changes in *S. sclerotiorum*’s transcriptome during pathogenic development in soybean, a differential expression analysis was performed between early and late infection in the S line. For the purpose of this analysis, “early infection” was defined as the average expression between 24 and 48 hpi, and “late infection” was defined as 96 hpi. These assignments were made because of the phenotypic differences between these timepoints (i.e. 24 and 48 hpi = restricted to inoculated petiole, 96 hpi = colonization of the main stem), but also due to a clear regulatory shift in gene expression between these stages of infection (Fig. 3). Differentially expressed genes (DEG’s) (False discovery rate (FDR) < 0.05; Average Reads Per Kilobase of transcript, per Million mapped reads (RPKM) > 1; and log_2_ fold change (logFC) ≥ 1 or ≤ − 1) were generated and categorized using BlastGo annotation and their predicted role in the infection cycle.

**Fig. 4:**
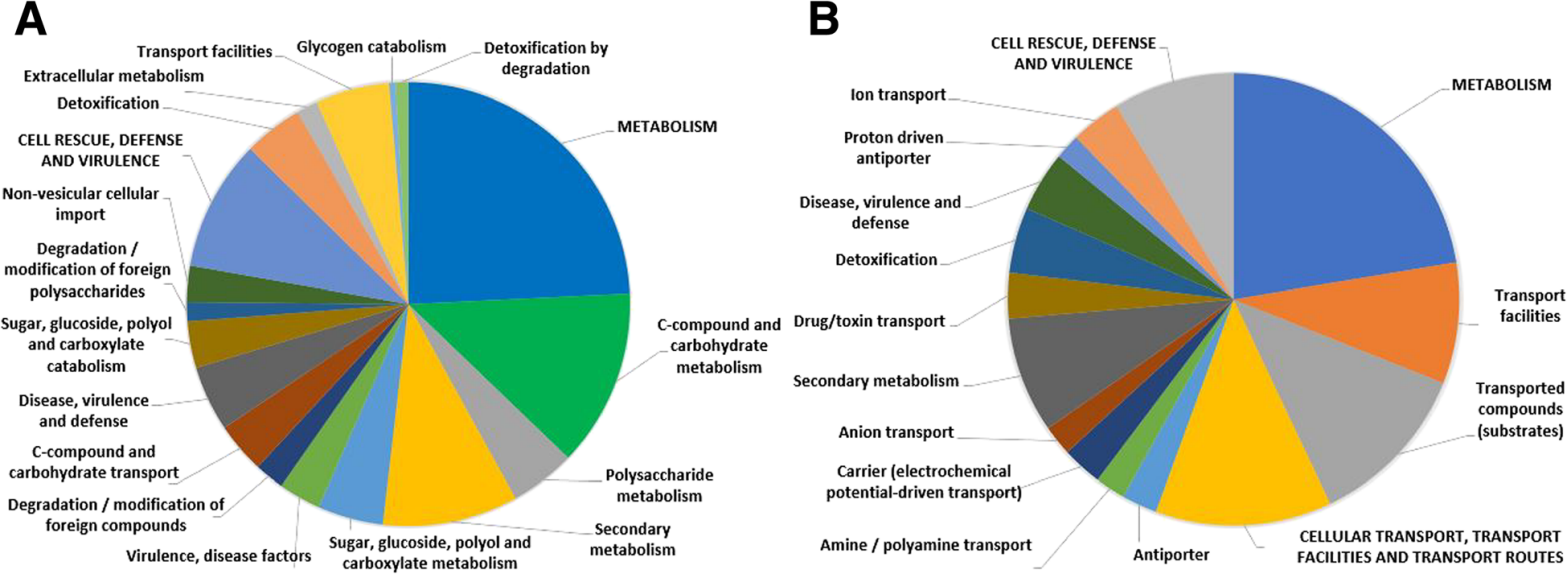
A functional distribution characterization of genes differentially expressed over the course of infection in the S line. **a** the characterized functional distribution of genes up-regulated during infection (Bonferroni correction < 0.05), **b** the characterized functional distribution of genes down-regulated during infection (Bonferroni correction < 0.05)

Six hundred fifty-five DEGs were identified over the course of the S line infection, with 76.1% (431/566) showing up-regulation late in infection (LATE) and 23.9% (135/566) showing the reverse trend with higher expression early in infection (EARLY) (Additional file 2: Table S2). A functional distribution characterization of both LATE and EARLY genes was performed using the functional distribution tool within the FunCat database (FunCatDb) and *S. sclerotiorum*’s annotated proteins (p3_r20263_Scl_scler) [31]. When functional categories were filtered to reflect only groups that were significantly enriched (Bonferroni correction < 0.05), a wide deviation was apparent. Of the significantly enriched LATE gene categories, most were related to metabolism (27.8%), degradation (16.7%), or catabolism (11.1%), all processes important in necrotrophic pathogens for colonization, degradation, and detoxification during infection. Similarly, several gene categories related to virulence and disease (16.7%) were upregulated late in infection (Fig. 4a; Additional file 3: Table S3A). Unlike the LATE genes, a majority of the categories applied to EARLY genes (66.7%) and majority of the total EARLY genes categorized (51.6%) were related to transport. (Fig. 4b; Additional file 3: Table S3B). While it’s difficult to infer the exact function of these transporters during soybean infection, it’s well established that fungi utilize an array of membrane bound transporters to facilitate the efflux of fungicidal toxins [32]. We also observed differences in the specific categories of transporters that were differentially expressed (Additional file 4: Figure S1; Additional file 2: Table S2). Although major facilitator superfamily (MFS) transporters involved in sugar transport (87.5%) were upregulated largely late in infection, MFS transporters involved in drug resistance and efflux (61.5%) and ABC Transporters (66.6%) were more highly expressed during early stages of infection. This suggests that *S. sclerotiorum* may prioritize the expulsion of antifungal toxins early in infection, but shifts focus to the transport of carbohydrates at a later stage when sugars have been released from the hosts cell walls.

Broad gene categorizations are often helpful in interpreting large transcriptomic data sets, but the important roles of individual genes can often be lost within the global view. To address this, up- and downregulated genes were manually analyzed using Protein BLAST (blastp). The two LATE genes most differentially expressed over the course of infection (logFC = 7.3–7.9) were surprisingly not classical virulence genes, but development-specific fungal lectins referred to as *Ssp1* (Sscle10g079320) and *Ssa* (Sscle01g001830) (Additional file 2: Table S2). The precise function of these lectins is unknown, but both were found to be abundant in sclerotia, and their upregulation late in infection may indicate a shift towards sclerotial formation [33, 34]. Sixteen genes, including *Ssp1* and *Ssa*, which were up-regulated late during infection, were also identified in a previous proteomic analysis as highly abundant gene products in the liquid exudates of *S. sclerotiorum* sclerotia [35] (Additional file 5: Table S4). The overlap of these genes upregulated at 96 hpi and the abundance of their products in sclerotial exudates provides evidence that the pathogen is likely shifting focus at the later stages of infection from virulence/colonization to survival structure formation.

Three of the EARLY genes which are upregulated during early infection make up the critical components of nitrate metabolism in *S. sclerotiorum* (Additional file 2: Table S2). These genes, consisting of a nitrate transporter (Sscle02g013580) which transports nitrate into the cytoplasm, a nitrate reductase (Sscle01g005230) that converts nitrate to nitrite, and a nitrite reductase (Sscle02g016460) which converts nitrite to ammonia, are most highly expressed during early infection and show a steep reduction in expression by 96 hpi (logFC = − 2.25-3). The potential importance of nitrogen metabolism in early infection is similarly reflected in the early upregulation of *S. sclerotiorum*’s urea active transporter (Sscle05g043930) and urease (Sscle05g047190) as well. This early expression pattern may be the result of relatively low nitrogen availability for the pathogen following penetration.

### *S. sclerotiorum* genes differentially regulated *in-planta*

**Fig. 5:**
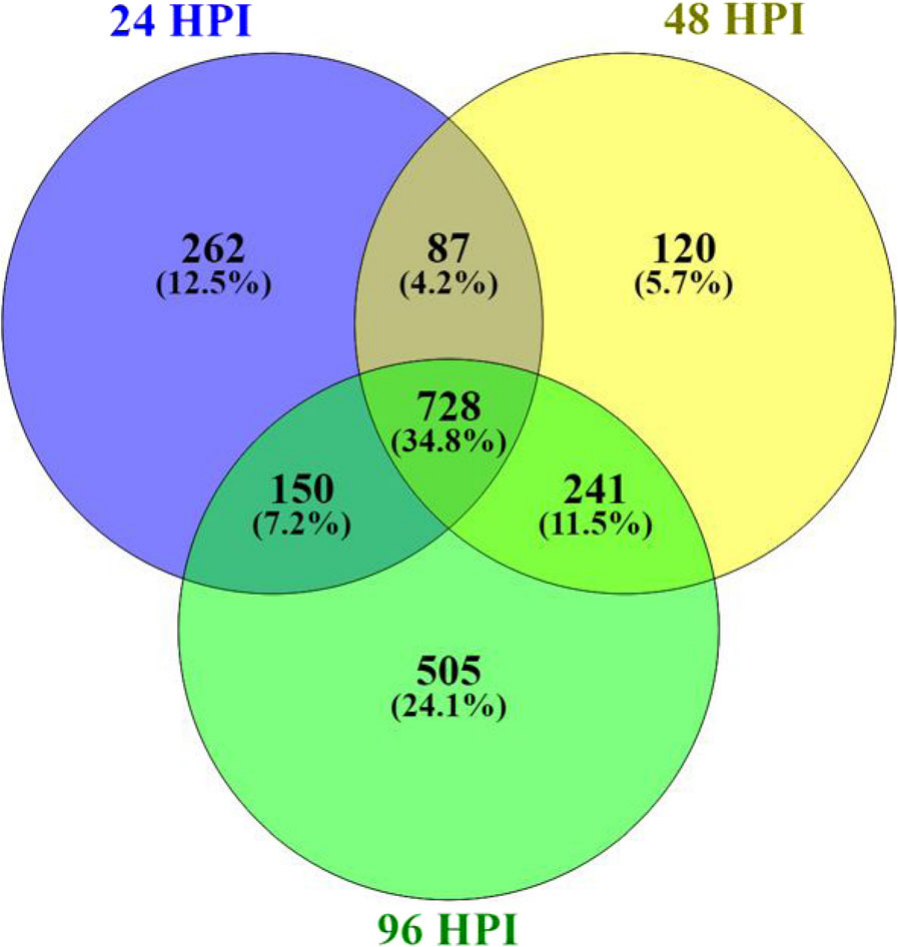
Venn diagram of genes up-regulated at each timepoint of infection (24, 48, and 96 hpi) when compared to the culture control (C)

To identify important pathogenicity components in *S. sclerotiorum*, a comparative expression analysis was performed between each timepoint of the S line infection (24, 48, and 96 hpi) and the culture control (C). DEG’s (False discovery rate (FDR) < 0.05; Average RPKM > 1; and log_2_ fold change (logFC) ≥ 1 or ≤ − 1) were generated and categorized using BlastGo annotation and their predicted role in the infection cycle. To focus on genes specifically induced during soybean infection, only genes with at least one time-point containing a logFC greater than one were considered. Over all three timepoints of infection, a total of 2093 putative genes showed positive regulation *in-planta* (Additional file 6: Table S5). Of these genes, 262, 120, and 505 were up-regulated exclusively at 24, 48, and 96 HPI, respectively, and 728 were up-regulated at all timepoints (Fig. 5).

### Cell wall degrading (CWD) and other hydrolytic enzymes

The successful degradation of plant cell walls is a pivotal step in both the colonization of host plants and in the release of sugars to be metabolized by fungal pathogens. *S. sclerotiorum*’s genome contains numerous genes that code for CWDEs [23], though, only a portion of this repertoire appears to be positively regulated during infection of soybean (Table 2).

**Table 2:** Hydrolytic enzymes up-regulated *in-planta*

Substrate	Hydrolytic Enzymes	DEGs in Category	Total Genes in Category	% Up-regulated
Lipids/Cutin	Lipid Degradation	33	46	71.7%
Polysaccharides	Pectin Degradation	29	32	90.6%
	Cellulose Degradation	26	46	56.5%
	Hemicellulose Degradation	28	28	100.0%
	Starch Degradation	5	8	62.5%
	Mannan Degradation	10	18	55.6%
	Callose Degradation	1	2	50.0%
	Arabinogalactan Degradation	9	10	90.0%
Proteins/Peptides	Protein Degradation	34	73	46.6%

The hydrolytic enzymes up-regulated at some time point of infection. Enzymes were putatively assigned to specific substrates based on Blast2GO annotation or characterized homologues

Of the CWDEs, those showing pectinolytic activity, such as endo- or exo-polygalacturonases (PGs), have garnered particular attention given their demonstrated importance in virulence across a range of fungal pathogens [19, 36, 37]. *S. sclerotiorum*’s genome contains nine previously identified PGs: *Sspg1* (Sscle16g108170), *Sspg2* or *Sspg1b* (Sscle03g022740), *Sspg3* (Sscle09g070580), *Sspg5* (Sscle02g018820), *Sspg6* (Sscle12g088720), *Ssxpg1* (Sscle02g018610), *Ssxpg2* (Sscle04g035440), and two uncharacterized putative exo-polygalacturonases (Sscle05g046840 and Sscle05g040500) [19, 20]. The upregulation of all 9 putative *S. sclerotiorum* PGs can be seen as the infection progresses, with the exception of *Sspg1*, *Sspg3*, and Sscle05g046840, which are expressed consistently at all timepoints (Average logFCs = ~ 8, ~ 5.5, and ~ 4.5, respectively), all other PGs show the greatest upregulation at 96 HPI (Additional file 7: Table S6).

During *S. sclerotiorum* infection on many hosts, the cuticle constitutes the first physical barrier to pathogen invasion. Given the nature of the cut-petiole inoculation technique used in this study, cutinase activity may not be required. However, most of the putative cutinases were either consistently induced at relatively high levels throughout infection (Sscle11g084380 and Sscle15g102320) or most induced at 96 HPI after the infection reached the main stem (Sscle02g011950) (Additional file 7: Table S6). Whether this is due to a consistent need to form lesions on the surface of the plant or a secondary purpose, such as interactions with other hydrophobic lipid substrates during colonization is unknown.

Proteases are hydrolytic enzymes which have been shown to act as important virulence factors in a variety of fungal plant pathogens through the degradation of proteins involved in the immune response of the host [38]. A total of 34 proteases were upregulated during this study (Additional file 7: Table S6). During early infection, a previously characterized non-aspartyl acid protease (*acp1*; Sscle11g082980) was found to be the most upregulated (logFC = 6.22 at 24 hpi) [39]. The largest group of proteases induced during infection were serine proteases, which were categorized as either carboxypeptidases, subtilisin-like proteins, or tripeptidyl peptidases, the latter two of which both belong to the subtilisin superfamily. Serine proteases have been characterized as virulence determinants in a large number of phytopathogenic fungi [40–42] and of the 17 serine proteases identified in this analysis, all 17 showed the highest expression at the initial stages of infection (24 hpi). Two other proteases were also upregulated during infection and are homologous to proteases involved in pathogenesis. An aspartyl (acid) protease, Sscle04g035550, was upregulated at all timepoints and was found to contain a SAPs domain (cd05474) commonly associated with plant pathogenic protease activity [43]. Another aspartate protease, Sscle07g058540, was the second most upregulated protease at 24 hpi and shows homology to several aspergillopepsin-like proteins (cd06097) whose activity is important in invasive aspergillosis of humans [44] (Additional file 7: Table S6).

### Secondary metabolite synthesis and detoxification

Many genes related to secondary metabolite (SM) synthesis and detoxification were up-regulated during at least one time-point of the infection. Although the function of a vast majority of these SMs is unknown, extensive study of *S. sclerotiorum*’s close relative *Botrytis cinerea* as well as other fungal pathogens have characterized many as phytotoxic and/or important in pathogenesis [45, 46]. Polyketide synthase (PKS) and Non-ribosomal peptide synthase (NRPS) appear to be the major enzymes associated with SM synthesis in *S. sclerotiorum*, and make-up 66.6 and 22.2%, respectively, of the SM synthesis genes found to be positively regulated in this study. Two PKSs (*SsPKS4*: Sscle05g048020 and *SsPKS11*: Sscle15g105000) were expressed to higher levels at all timepoints after inoculation, as compared to the in vitro control. Two other PKSs (*SsPKS10*: Sscle04g032810 and *SsPKS15*: Sscle14g097860), which saw no induction during the infection of *B. napus* [24], were highly up-regulated (logFC = ~ 4) at 24 HPI, indicating a possible role in early infection of soybean. A single phytoene synthase (*SsPHS1*: Sscle02g017510) was also upregulated at all timepoints (Additional file 8: Table S7).

Although PKSs and NRPSs are the primary genes involved in SM synthesis, cytochrome P450s (CYP450s) play a pivotal role in both the biosynthesis of fungal mycotoxins and the detoxification of host metabolites through the conversion of potentially toxic compounds to more hydrophilic derivatives [47, 48]. A large number of CYP450s were positively regulated at some timepoint of the soybean infection, with 16.2% (6/37) and 37.8% (14/37) of the genes showing up-regulation at only 24 and 96 hpi, respectively. The most up-regulated CYP450 at 24 and 48 hpi was Sscle04g033880, a gene showing homology to pisatin demethylase in *Aspergillus lentulus* (Additional file 9: Table S8). Another CYP450, Sscle08g067130, was highly induced throughout infection of both *B. napus* [24] and in this study in soybean, indicating a potentially conserved requirement during infection of both crops, whereas Sscle05g045810, which was highly upregulated at all timepoints during this study, upregulation of this gene was not observed during *B. napus* infection*.* [24] (Additional file 9: Table S8).

**Fig. 6:**
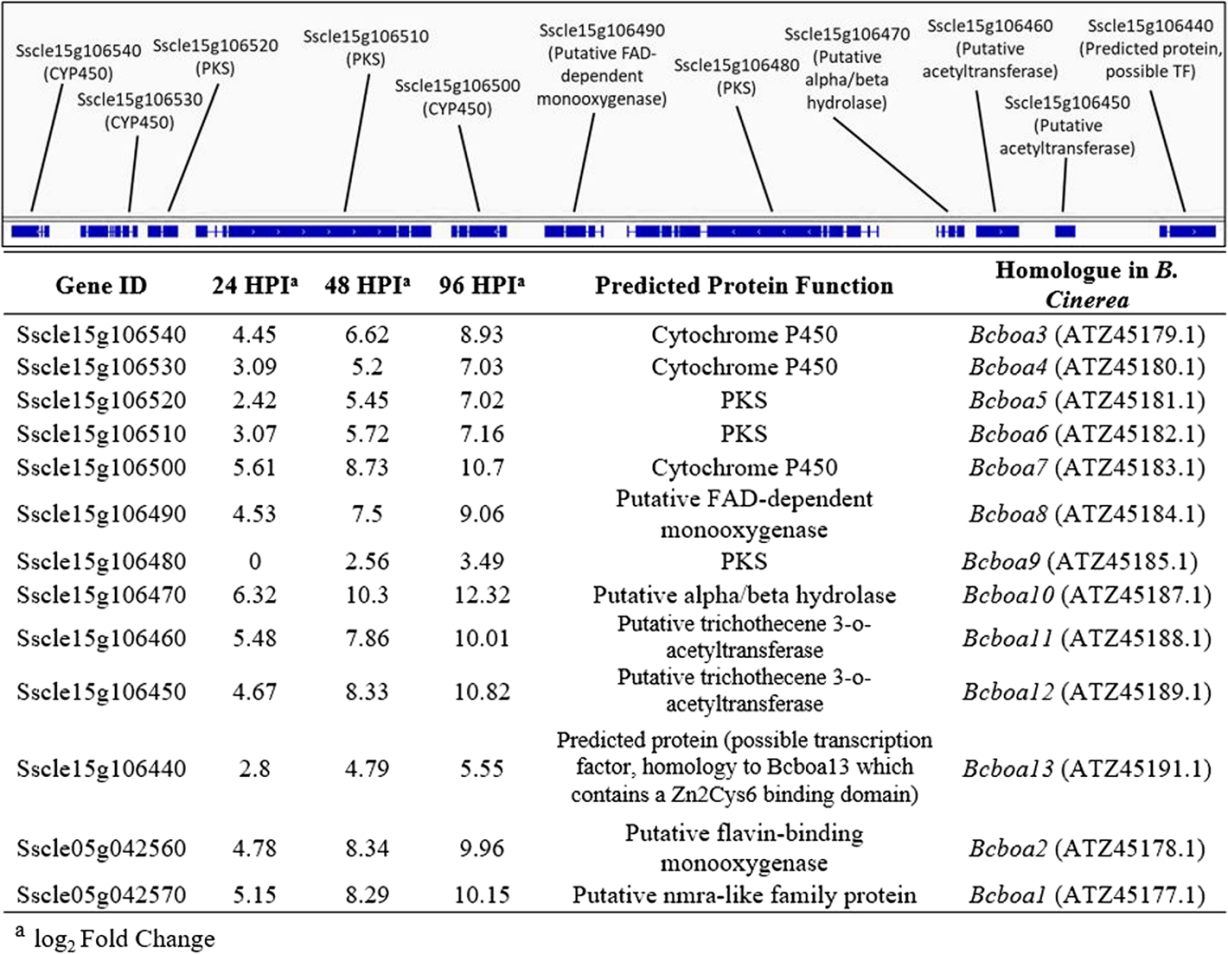
Secondary metabolite cluster identified on chromosome 15 of *S. sclerotiorum*. The cluster contains three putative Cytochrome P450s (Sscle15g106540, − 30, − 00), three putative PKSs (Sscle15g106520, − 10, − 480), two putative acetyltransferases (Sscle15g106460 and − 50), a putative FAD-dependent monooxygenase (Sscle15g106490), a putative alpha/beta hydrolase (Sscle15g106470), and a predicted protein of unknown function (Sscle15g106440). The gene loci, logFCs (compared to culture), predicted function, and homologue in *B. cinerea* of each gene are shown. Sscle05g042560 and Sscle05g042570 appear within this gene cluster in *B. cinerea*, but are found on chromosome 5 of *S. sclerotiorum*

Botcinic acid is an important virulence factor of *B. cinerea*, and despite finding the orthologous SM cluster in *S. sclerotiorum*, previous studies have failed to identify the toxin in *S. sclerotiorum* [49] or the upregulation of the pivotal PKS genes *in-planta* [24]. However, we found all 13 orthologs of the botcinic acid gene cluster (*Bcboa1–13)* to be upregulate in this study, with all showing the highest upregulation at 96 hpi (Fig. 6). Nearly all of the most upregulated SM synthesis genes and CYP450s belong to this cluster, including the two PKS genes directly implicated in botcinic acid biosynthesis (Sscle15g106510 and Sscle15g106480), an additional PKS (Sscle15g106520), a putative FAD-dependent monooxygenase (Sscle15g106490), and three CYP450’s (Sscle15g106540, Sscle15g106530, and Sscle15g106500).

**Fig. 7:**
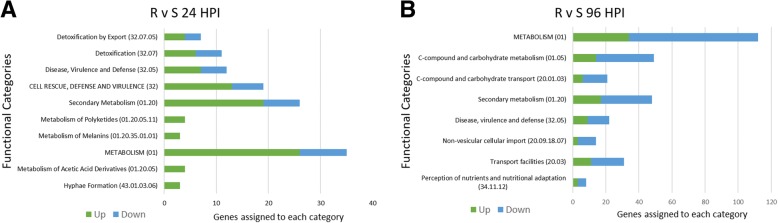
Functional distribution of differentially expressed in the R and S lines at 24 (**a**) and 96 hpi (**b**). Green regions of each bar represent genes which were up-regulated in the R line. Blue regions of each bar represent genes which were down-regulated in the R line

Along with the multifaceted role of CYP450’s in SM metabolism, fungal pathogens require a suite of other enzymes to detoxify the fungicidal metabolites released by their hosts. This can be achieved through enzymatic modification of the metabolites or through the activity of transporters which can prevent the accumulation of harmful toxins [32]. By far the most upregulated detoxification enzyme at all timepoints was the brassinin glucosyltransferase *Ssbgt1* (Sscle01g003110; logFC = 7.2–8.8). *Ssbgt1*is expressed in response to the production of a variety of plant phytoalexins in the host, and was shown to specifically degrade the antimicrobial compound brassinin via glucosylation [50]. *Ssbgt1* was observed to be the most upregulated detoxification enzyme during the *S. sclerotiorum* infection of *B. napus* as well, indicating a strong phytoalexin response in both hosts [24] (Additional file 6: Table S5).

Laccases are a class of copper-containing oxidase enzymes that are known to oxidize a wide range of phenols as their substrates [51]. A recent analysis of the *S. sclerotiorum*-soybean pathosystem identified phenolic compounds generated in the phenylpropanoid pathway as a pivotal component of soybean resistance, thus highlighting a potentially critical role for laccases in suppressing this immune response [30]. Only two laccases, a putative laccase 2 (Sscle03g023030) and a probable laccase precursor (Sscle13g092370), were upregulated in comparison to culture, the latter of which was only upregulated at 48 hpi. The former laccase gene, Sscle03g023030, is notable both because of its impressive upregulation *in-planta* and because it was not documented in the transcriptomic analysis of *B. napus* [24] or *P. vulgaris* [25]. As well as being up-regulated at all timepoints, Sscle03g023030 is among the most differentially expressed between early and late infection, with its greatest upregulation at 24 hpi (logFC = 7.8) and lowest at 96 hpi (logFC = 2.8) (Table 3).

**Table 3:** Detoxification enzymes up-regulated in-planta

Gene ID	24 HPI^a^	48 HPI^a^	96 HPI^a^	Blast2GO Description
Sscle03g027760	1.98	1.7	1.83	2-nitropropane dioxygenase
Sscle12g087340	–	–	1.93	2-nitropropane dioxygenase
Sscle11g085500	–	–	1.13	2-nitropropane dioxygenase protein
Sscle01g007130	2.71	3	4.64	carbon-nitrogen hydrolase
Sscle08g067590	–	–	1.66	glutathione s-transferase
Sscle06g051110	1.31	1.09	1.06	glutathione s-transferase
Sscle10g075830	–	1.44	1.74	glutathione s-transferase
Sscle15g104750	5.06	4.73	6.36	glutathione s-transferase
Sscle06g053300	1.39	1.64	1.93	glutathione s-transferase kappa 1
Sscle01g005000	3.8	3.43	3.29	glutathione transferase
Sscle01g003110	7.17	8.66	8.79	udp-glucosyltransferase (brassinin)
Sscle03g023030	7.08	4.84	2.83	laccase 2
Sscle13g092370	-	3.65	-	multicopper oxidase

^a^log_2_ Fold Change

The logFC and Blast2GO description of genes putatively assigned as detoxification enzymes within S. sclerotiorum. LogFC values were generated through the comparison of individual timepoints of the S line infection and the culture control

Glutathione S-transferases (GSTs) are known for their ability to detoxify a wide range of toxic compounds through glutathione conjugation [52]. Six GSTs were upregulated in this analysis compared to culture conditions (Sscle01g005000, Sscle08g067590, Sscle06g051110, Sscle10g075830, Sscle15g104750, Sscle06g053300), forming the largest group of detoxification enzymes induced during infection. Compared to other detoxification enzymes, these six GSTs were expressed at relatively consistent levels throughout infection, potentially indicating a constant pressure from plant phytotoxins throughout infection (Table 3).

This pattern is largely conserved within the three 2-nitropropane dioxygenase genes (Sscle03g027760, Sscle12g087340, and Sscle11g085500) which constitute the second largest group of detoxification enzymes upregulated *in-planta* (Table 3).

As previously discussed, transporter proteins, especially within the ABC transporter class, can play an important role in the efflux of fungicidal phytotoxins during infection [53, 54]. Of the eight putative ABC transporters up-regulated during infection, three (Sscle01g004040, Sscle02g016430, Sscle14g097690) were among the most up-regulated transporters at any timepoint and all were most induced during early infection (Additional file 10: Table S9). The most up-regulated of those three, Sscle01g004040, shares high homology (88%) to *Bmr3*, a transporter characterized in *B. cinerea* and found to be strongly induced following exposure to a variety of fungicidal toxins, including mepanipyrim, the grapevine phytoalexin resveratrol, and two dicarboximides [55]. The second, Sscle02g016430, encodes a homolog to *Bmr1*, another ABC transporter closely related to *Bmr3*, and was most highly expressed at 48 hpi. The third, Sscle14g097690, is a homolog of the *B. cinerea* gene *BcatrB*, known to be important in fungal tolerance to the phytoalexins camalexin and resveratrol [54, 56]. Sscle14g097690 shared the same expression pattern as the other two, demonstrating the greatest upregulation at 48 hpi (Additional file 10: Table S9). The upregulation of these various mechanisms of detoxification indicates a strong phytoalexin pressure during *S. sclerotiorum* infection of soybean, which agrees with previous work highlighting the production of antifungal compounds in soybean during SSR infection [30].

### Transcription factors

Transcription factors (TFs) are proteins containing at-least one DNA-binding domain (DBD) that act as pivotal regulators of gene expression throughout the lifecycle of all Eukaryotes. Because of the potentially positive or inhibitory role that TFs can have on the pathways that they regulate, all DEG’s (logFC > 1 or < − 1) were considered for this analysis, as opposed to only those showing positive regulation. Although many TFs have been annotated, only *Ssfkh1* (Sscle06g049780), *Ssfoxe2* (Sscle05g046830), and *Pac1* (Sscle06g049830) have been functionally characterized in *S. sclerotiorum* [8, 57, 58]. Of the 197 putative TFs identified in the *S. sclerotiorum* genome, 68 appear to be differentially regulated during infection (~ 34.5%) (Additional file 11: Table S10). Interestingly, neither *Ssfkh1* or *Pac1* is differentially expressed, despite their demonstrated role in virulence. Two zinc finger containing TFs very close to one another on chromosome 16 (Sscle16g110450 and Sscle16g110180) and another on chromosome 9 (Sscle09g071930) are the most differentially regulated TFs at 24 HPI, with logFCs of − 5.12, − 3.43, and − 3.02, respectively (Additional file 11: Table S10).

### Putative secreted effectors

Unlike their filamentous cousins, the oomycetes, true fungi lack any conserved domains, such as the RXLR motif, to help in the identification of their effectors [59]. However, other factors were utilized in previous studies to single out putative effector proteins in *S. sclerotiorum* [22, 60].

Of the 4602 DEGs identified in this study, 57 were identified as putative secreted effectors. Of these 57 genes, only 18 were differentially regulated during the course of infection (early v. late) (Table 4). The large number of putative effectors that are not differentially expressed over the course of infection could point to a more constitutive expression of effectors in *S. sclerotiorum* than is commonly seen in biotrophic and hemibiotrophic fungi, which often alter effector expression as they transition between stages of infection (i.e. appressoria formation, penetration of invasive hyphae, differentiation into necrotrophic hyphae) [61–63].

**Table 4:** Putative secreted effectors up-regulated during infection

Gene ID	24 HPI	48 HPI	96 HPI	Dis. Progress	Notes/Citations
Sscle03g028510	12.19	10.01	6.15	−4.98	Possible virulence factor characterized in *B. napus* infection (Ssv263) [15].
Sscle07g055350	8.03	8.87	10.83	–	Contains a CFEM domain (pfam05730). Found to be up-regulated in Brassica infection [24]
Sscle05g045060	8.4	8.84	9.09	–	Identified as a predicted effector by Derbyshire et al. 2017 [58].
Sscle06g048920	8.68	9.33	8.24	–	Identified as a predicted effector by Derbyshire et al. 2017 [58].
Sscle06g055310	7.72	8.02	7.76	–	Related to Immunoglobin E (IgE) binding proteins in Aspergillus fumigatus (~ 40% identity). Homologue to Sscle11g080900 (99% identity).
Sscle11g080900	7.25	7.64	7.37	–	Related to Immunoglobin E (IgE) binding proteins in Aspergillus fumigatus (~ 40% identity). Homologue to Sscle06g055310 (99% identity).
Sscle16g107670	6.97	6.68	7.15	–	A gene containing a cerato-platanin domain (pfam07249) which was characterized as *Sscp1* and found to be important in full virulence of *S. sclerotiorum* [62]
Sscle08g068200	6.29	7.05	6.52	–	Contains two chitin recognition domains (pfam00187) and a fungal cellulose binding domain (pfam00734).
Sscle01g003850	4.96	6.03	7.48	1.98	Characterized as a virulence gene in *S. sclerotiorum* (*Ssssvp1*). Binds with a subunit of the cytochrome b-c1 complex of the mitochondrial respiratory chain and prevents subcellular localization into the mitochondria, subsequently leading to plant cell death [18].
Sscle12g090490	5.17	5.78	5.9	–	A necrosis and ethylene-inducing peptide (SsNEP2) found to induce cell death in plants [16].
Sscle05g041050	3.43	5.52	7.66	3.19	Identified as a predicted effector by Derbyshire et al. 2017 [58].
Sscle03g024000	5.15	4.72	4.81	–	Shares 59% identity with *Colletotrichum hingginsianum* effector candidate 91 (CHEC91) [104] and a *Colletotrichum incanum* gene categorized as a “hypersensitive-response inducing protein.”
Sscle08g064180	4.29	5.26	4.97	–	Identified as a predicted effector by Derbyshire et al. 2017 [58].
Sscle03g022790	2.69	5.47	5.7	–	Contains a fungal hydrophobin domain (pfam06766). Possibly involved in mediating the interaction of fungal tissue and the plant. Often connected to the outside of fungal conidia or on the hyphal wall.
Sscle11g082980	6.22	3.86	3.57	–	A non-aspartyl acid protease (acp1) found to be expressed during S. sclerotiorum infection of sunflower cotyledons. [38]
Sscle06g049430	6.57	3.11	3.72	–	A secretory Rhs repeat-containing protein (Ss-Rhs1) found to be important in sclerotial development and hyphal infection. [87]
Sscle04g040080	3.49	3.76	5.08	–	Identified as a predicted effector by Derbyshire et al. 2017 [58]. Contains a protein domain of unknown function (DUF3129; pfam11327).
Sscle08g063910	3.95	3.14	4.18	–	Found to be up-regulated in Brassica infection [24].
Sscle07g061770	–	4.19	5.11	–	Identified as a predicted effector by Derbyshire et al. 2017 [58].
Sscle10g075140	–	5.37	3.72	–	Identified as a predicted effector by Derbyshire et al. 2017 [58].
Sscle06g052360	3.52	2.63	2.82	–	Identified as a predicted effector by Derbyshire et al. 2017 [58].
Sscle15g106630	4.34	2.76	1.76	−1.82	Found to be up-regulated in Brassica infection [24].
Sscle16g111080	3.77	2.73	1.71	−1.56	A putative chorismate mutase which is expressed in S. sclerotiorum infection of Brassica and is predicted to interfere with salicylic acid (SA) signaling in infected plants [88].
Sscle05g042890	2.31	2.76	2.51	–	Contains a Lytic polysaccharide mono-oxygenase, cellulose-degrading domain (LPMO-10; pfam03067), related to cellulose degradation. This domain was previously characterized as a chitin binding domain.
Sscle01g000190	2.38	2.4	2.61	–	Contains a pyrroline-5-carboxylate reductase domain (pfam14748). Possibly involved in proline biosynthesis.
Sscle16g107890	3.21	3.58	–	–	Identified as a predicted effector by Derbyshire et al. 2017 [58].
Sscle07g062010	1.38	2.82	2.29	–	Contains a glycosyl hydrolases, family 18 (GH18) type II chitinase. Found to be up-regulated in *B. napus* infection [24]
Sscle07g058780	1.44	2.2	2.18	–	Found to be up-regulated during Arabidopsis infection [105].
Sscle04g035160	–	2.69	3.1	–	Identified as a predicted effector by Derbyshire et al. 2017 [58].
Sscle13g096120	3.42	–	2.07	–	Identified as a predicted effector by Guyon et al. 2014 [22].
Sscle08g065470	2.24	1.56	1.66	–	Contains an amidase domain (pfam01425). Identified as a predicted effector by Guyon et al. 2014 [22].
Sscle06g054400	–	1.72	3.11	–	Identified as a predicted effector by Derbyshire et al. 2017 [58].
Sscle10g078230	–	3.05	1.71	–	A putative salicylate hydrolase. Contains salicylate 1-monooxygenase (TIGR03219) and FAD binding (pfam01494) domains.
Sscle08g064590	–	2.17	2.53	–	Identified as a predicted effector by Derbyshire et al. 2017 [58].
Sscle12g088370	–	1.43	3.16	2.6	Identified as a predicted effector by Guyon et al. 2014 [22].
Sscle06g054180	–	–	4.37	4.53	Contains a Lysin motif (smart00257). Found to be up-regulated in Brassica infection [24].
Sscle07g057820	1.52	1.27	1.31	–	A likely subtilisin-like serine protease. Also contains a peptidase inhibitor domain (pfam05922), suggesting that it may be at the very least a temporary protease inhibitor.
Sscle06g050820	2.08	–	1.96	–	Identified as a predicted effector by Derbyshire et al. 2017 [58].
Sscle07g062060	1.19	1.32	1.46	–	Identified as a predicted effector by Derbyshire et al. 2017 [58].
Sscle01g007640	1.2	1.23	1.22	–	A serine protease inhibitor containing a Peptidase inhibitor I9 domain (PF05922) which was expressed during infection of Arabidopsis [22].
Sscle14g100310	–	–	3.56	3.54	Identified as a predicted effector by Derbyshire et al. 2017 [58].
Sscle01g005870	–	–	3.12	1.79	Putative Choline dehydrogenase or related flavoprotein (COG2303). Potentially involved in lipid transport or metabolism. 36% identity to Sscle06g048860.
Sscle08g066850	–	–	2.4	3.22	Contains chitin recognition domain (pfam00187) and GH18 domains containing both catalytically active and inactive chitinase-like proteins (cd02872).
Sscle14g098710	–	2.3	–	–	. Contains a Common central domain of tyrosinase (pfam00264).
Sscle08g068500	–	–	2.06	–	Characterized as *Ssitl* and found to suppress JA signaling pathways
Sscle01g000660	2.19	–	–	–	Identified as a predicted effector by Derbyshire et al. 2017 [58].
Sscle04g036550	2.15	–	–	–	Identified as a predicted effector by Derbyshire et al. 2017 [58].
Sscle01g004200	–	–	2.16	–	Homologue of the predicted effector 5 protein discovered in *Venturia inaequalis* (Thakur et al. 2012).
Sscle14g098880	–	–	1.94	2.99	Contains a ricin type beta-trefoil lectin domain (PF00652). This domain is common in the formation of toxins, but is also known to be involved in signal transduction.
Sscle04g038020	–	–	1.84	5.3	Contains a CyanoVirin-N Homology (CVNH) domain (pfam08881). Experimentally confirmed to be essential for full virulence [106].
Sscle12g090380	–	1.84	–	–	Identified as a predicted effector by Derbyshire et al. 2017 [58].
Sscle06g050280	–	–	–	5.04	Contains a CFEM domain (pfam05730) often associated with fungal pathogenesis. Up-regulation was observed in *B. napus* infection, but not during the infection of several other host plants [22, 24].
Sscle06g048860	–	–	–	3	Putative Choline dehydrogenase or related flavoprotein (COG2303). Potentially involved in lipid transport or metabolism. 36% identity to Sscle01g005870.
Sscle08g063080	–	–	–	1.87	Putative tripeptidyl peptidase shown to be up-regulated during infection of *B. napus* [24].
.Sscle06g053560	–	–	–	2.5	Shares 72% identity with a cuticle-degrading serine protease found in *Marssonina brunnea.* Also contains a peptidase inhibitor domain (pfam05922).
Sscle02g012940	–	–	–	2.68	Identified as a predicted effector by Derbyshire et al. 2017 [58].
Sscle01g008950	–	–	–	2.75	Identified as a predicted effector by Derbyshire et al. 2017 [58], but found to be strongly up-regulated in developing apothecia. The relationship between the gene and virulence is considered suspect [107].

All putative secreted effectors identified as either up-regulated in-planta or differentially regulated over the course of infection. Justification for each gene’s inclusion was given either through the description of previous characterization, relevant conserved domains/homology, or the study which predicted its candidacy.

The previously characterized *Ssv263* (Sscle03g028510) [15] has the largest logFC of any putative effector compared to culture conditions (24 hpi = 12.19, 48 hpi = 10.01, and 96 hpi = 6.15) and early v. late infection (log FC = − 5.32), suggesting its likely importance earlier in the infection process. This was also true for a Rhs repeat-containing protein (*Ss-rhs1*; Sscle06g049430) (71), found to be important for sclerotial development and hyphal infection (Table 4). *Ssnep2* (Sscle12g090490), which was characterized as necrosis and ethylene-inducing peptide on *Nicotiana benthamiana* [16], was up-regulated at all timepoints of the infection (Table 4). *Ssssvp1* (Sscle01g003850) and *Sscp1* (Sscle16g107670), two fungal proteins shown to induce programmed cell death in plants [18, 64], were highly upregulated at all timepoints in comparison to culture, but showed peak expression at 96 hpi (Table 4). The *S. sclerotiorum* integrin-like gene (*Ssitl*; Sscle08g068500), a secreted effector known to suppress jasmonic/ethylene (JA/ET) signaling [17], was only upregulated as compared to culture at 96 hpi (Table 4).

Two uncharacterized genes encoding CFEM domain-containing proteins were also identified in this study, the first of which (Sscle07g055350) was among the most upregulated during infection, with logFCs > 8 at all timepoints, while the second (Sscle06g050280) showed strong induction at the later stages of infection compared to early (logFC = 5.04).Two genes with no conserved domains and with no known nor characterized homology to other effectors, Sscle05g045060 and Sscle06g048920, were the third and fourth most upregulated at 24 hpi, respectively, and demonstrated consistently high expression throughout infection. Two other highly upregulated genes, Sscle06g055310 and Sscle11g080900, share 99% homology with one another and are closely related to an Immunoglobin E (IgE) binding proteins in *Aspergillus fumigatus* (~ 40% identity) (Table 4).

To avoid detection and protect their cell walls from enzymatic degradation, many fungal pathogens have evolved strategies to bind their stray chitin to avoid the induction of plant pattern triggered immunity (PTI) and/or plant chitinase activity [65]. Three genes putatively in this category (Sscle08g068200, Sscle07g062010, and Sscle08g066850) contain chitin binding domains and were up-regulated in at-least one timepoint, although whether these genes are acting purely as chitin binding proteins or as chitinases involved in fungal cell wall modification is unknown. Sscle08g068200 and Sscle07g062010 were up-regulated at all timepoints of infection, while Sscle08g066850 was upregulated only at 96 hpi (Table 4).

### Regulation of oxalic acid (OA) and reactive oxygen species (ROS)

OA is a key virulence factor for *S. sclerotiorum* that is responsible for the upregulation of ROS within the host, and eventually the induction of programmed cell death (PCD) to the benefit of the pathogen [13, 66]. Thus, redox regulation during pathogenic development is likely to be critical to both the pathogen and the host.

The most well studied gene in OA biosynthesis is and oxaloacetate acetylhydrolase (*Ssoah1*; Sscle10g075560) which catalyzes the conversion of oxaloacetate to OA and carbon dioxide [67]. Previous studies in *Phaseolus vulgaris* and *Brassica napus* showed an up-regulation of *Ssoah1* during infection [24, 25]. Surprisingly, this pattern was not conserved in the infection of soybean, and *Ssoah1* showed no upregulation over the course of infection or when compared to culture (Tables S2 and S5). To confirm that *Ssoah1* was being expressed during infection, an analysis of the average expression of each gene was conducted on all timepoints and reported as the average log_2_ of the read counts per million (Ave. Exp.) (Additional file 12: Table S12). This examination revealed that of the 9161 genes with an Ave. Exp. > 1, *Ssoah1* was the sixth highest (Ave. Exp. = 12.5), and was excluded from the differential expression analysis because it was expressed at similar levels both in vitro and *in planta* (Additional file 13: Table S11). Given that media acidification is commonly used as a measure of OA biosynthesis within *S. sclerotiorum*, the expression of *Ssoah1* in culture is not unexpected [67, 68]. This analysis also highlighted two other genes with high average expression, the peroxiredoxin (Sscle06g048910) and the alcohol oxidase (Sscle03g024060), which were found to be both upregulated *in planta* and have likely roles in redox regulation (Additional file 12: Tables S12 and Additional file 6: Table S5).

Previous work in *B. napus* indicated that expression levels of oxalate decarboxylase (Sscle09g069850), an enzyme known to degrade OA, were up-regulated in concert with *Ssoah1*, likely due to a careful balance in the regulatory pathways of OA needed for successful infection [24]. A similar pattern was also seen during infection of soybean, given that both genes fell between logFCs of − 1 and 1 over the course of infection and failed to qualify as a DEG, indicating constitutive expression.

NADPH oxidase (NOX) genes have been reported in a variety of species to be important ROS generators [66, 69, 70]. The two NOX genes identified in *S. sclerotiorum*, *Ssnox1* (Sscle05g048220) and *Ssnox2* (Sscle12g087830), were both found to be consistently expressed over the course of infection and *Ssnox2* showed very similar expression patterns between infection and culture. *Ssnox1*, in contrast, showed significantly reduced expression in-planta as compared to culture (logFC = − 2.47) (Additional file 6: Table S5).

While the induction of ROS in the plant is often the focus in this pathosystem, the ability of *S. sclerotiorum* to cope with ROS within the host is likely to be crucial. Along with the previously discussed peroxiredoxin, a Cu/Zn superoxide dismutase (SOD) gene (*Sssod1*; Sscle03g025030) was shown to be important for virulence [71]. Sscle03g025030 was upregulated throughout our time course compared to culture (Additional file 14: Table S13), confirming its importance during infection. Catalases and peroxidases can also serve as important ROS scavengers [72]. Four catalase enzymes (Sscle03g026200, Sscle04g037170, Sscle05g047950, and Sscle15g104430) were upregulated during infection, all showing highest expression at 96 hpi and no induction at 24 hpi (Additional file 14: Table S13), likely to coincide with peak ROS levels at the later stages of infection. The most upregulated catalase was the previously characterized *Scat1* (Sscle04g037170), though its role in ROS detoxification is unclear [73]. Of the four peroxidase genes upregulated when compared to culture, two (Sscle03g024750 and Sscle01g000730) show the greatest expression at 24 hpi and two (Sscle07g058850 and Sscle06g053530) show greatest expression at 96 hpi (Additional file 14: Table S13). Two additional peroxidases were not upregulated in comparison to culture, but did show either upregulation (Sscle09g070530; logFC = 2.02) or downregulation (Sscle01g007350; logFC = − 1.88) when comparing early and late infection (Additional file 2: Table S2). Overall, detoxification of ROS is clearly a priority throughout the course of infection, but becomes particularly critical during the later stages of when OA induced ROS production induces broad scale programmed cell death in the host.

### Gene expression during *S. sclerotiorum* challenge of R and S *Glycine max*

Transcriptomic analyses comparing R and S cultivars during infection with a pathogen have become an increasingly popular method of elucidating the mechanisms of plant resistance [30, 74﻿]. The characterization of differentially regulated plant genes, proteins, and pathways can be pivotal in understanding novel mechanisms of resistance, but the effect of plant resistance on pathogen gene expression can be provide insight into fungal mechanisms deployed to counter this resistance as well.

DEGs were analyzed between the R and S infections for each timepoint of infection (24, 48, and 96 hpi; *p*-value < 0.01; logFC > 1 or < − 1). 99, 62, and 351 DEGs were generated through the comparison of R and S at 24, 48, and 96 hpi, respectively (Additional file 15: Table S14). These genes were classified into broader categories using the functional distribution tool within the FunCat database (FunCatDb) and *S. sclerotiorum*’s annotated proteins (p3_r20263_Scl_scler) [31] (Additional file 16: Table S15). An interesting observation is that of the DEG’s at 24 hpi, 76.7% (76/99) were up-regulated in R line infection, as opposed to only 23.4% (82/350) which were upregulated at 96 hpi. The fact that the R line largely suppressed SSR infection of its main stem (Fig. 1) suggests that this difference at 96 hpi is likely due to a developmental delay of *S. sclerotiorum*.

Despite the two infections being out-of-sync at 96 hpi, the functional distribution analysis of the DEGs at 24 hpi revealed potentially important strategic differences in gene expression between the two infections. Work to characterize the nature of the 91–145 line resistance noted an increased production of fungicidal organic acids [30], and we concurrently expected that the fungus in the R line would positively regulate genes related to detoxification in order to compensate. Although some regulatory shifts of these genes are observed, a similar number of genes related to detoxification/toxin transport were up- and downregulated in the resistant infection as compared to the susceptible (Fig. 7a). The most marked result of this analysis was the up-regulation of genes related secondary metabolism, particularly melanin. So far, only *Scd1* (Sscle03g031470), *Thr1* (Sscle03g031480), and *Sspks13* (Sscle03g031520) have been characterized as genes pivotal in melanin biosynthesis within *S. sclerotiorum* [75], although a tetrahydroxynaphthalene reductase (Sscle09g070740) also contains homology to known components of this pathway in other fungi. All four of these genes were upregulated in the R infection at 24 hpi with logFC = ~ 2.0–2.4. Given that one of the most important protective characteristics of melanin is its role as a hydrophobe within the fungal cell wall, the remaining DEGs at 24 HPI were analyzed to identify any other positively regulated structural or surface proteins serving a similar purpose.

Three putatively hydrophobic cell surface proteins were found to have been up-regulated from this list. The first gene, Sscle15g106410, contains a fungal hydrophobin domain (pfam06766) which is a small, moderately hydrophobic extracellular molecule that typically binds to the outside of fungal hyphae to mediate contact and communication between fungi and the environment. The second gene, Sscle12g091650, contains a Hydrophobic surface binding protein A (HsbA) domain (pfam12296) which was identified originally in *Aspergillus oryzae* as a surface protein important in both the adhesion to and degradation of hydrophobic surfaces [76]. The third gene, Sscle09g070510, contains repeated fasciclin domains (pfam02469) which have been shown in *Magnaporthe oryzae* to be important in cell adhesion and binding to hydrophobic surfaces [77].

Prior work attempting to characterize the mechanism of SSR resistance within the R line indicated that it’s antimicrobial activity likely targeted the biosynthesis/stability of ergosterol, a crucial component of fungal cell walls, through an unknown mechanism [66]. To determine if this activity affected the transcription of genes important in the biosynthesis of ergosterol, or if the fungus within the R line might be overproducing ergosterol in an attempt to offset these effects, several genes known to be important in this pathway were analyzed. Neither Erg28 (Sscle11g086600), sterol 24-C-methyltransferase (Sscle07g056400), nor CYP51 (Sscle02g013950), all of which have been characterized as important components of ergosterol synthesis, were found to be differentially regulated between the two lines at any timepoint [78–80].

Other genes of interest which were up-regulated in this timepoint comparison include a salicylate hydroxylase (Sscle05g045180) and a well characterized oxalate decarboxylase (Sscle09g069850). Previous studies have demonstrated *S. sclerotiorum*’s capacity to degrade salicylate in-vitro and Sscle05g045180 was identified as an enzyme potentially responsible for this degradation [81]. Oxalate decarboxylase (Sscle09g069850) is known to degrade OA, a pivotal virulence component during *S. sclerotiorum* infection. Although this activity appears counterintuitive given OA’s importance in infection, the work characterizing this gene showed its induction was important in the formation of compound appressoria for host penetration [82]. This, along with the upregulation of the previously discussed hydrophobic surface proteins suggests that some expression differences at 24 hpi may be related to differences in *S. sclerotiorum*’s penetration of the R and S lines.

## Discussion

As a broad-host range, predominantly necrotrophic pathogen, *S. sclerotiorum* has a range of virulence factors, including CWDEs, detoxification enzymes, secreted effectors, and metabolites, that it employs to successfully infect a plethora of hosts [26]. Studies have singled out specific factors in the pathogenic development of *S. sclerotiorum*, largely in model plants [83]. However, only a few studies have focused on individual *S. sclerotiorum*-host interactions in crop plants. In this study, we provide a global overview of transcript regulation in *S. sclerotiorum* in association with soybean, and identify stage-dependent biological processes utilized by this fungus to successfully infect this important crop.

The temporal regulation of gene expression in culture, and in the early stages of infection compared to late infection highlighted the potential importance of transporters in the toxin efflux process during fungal infection. ATP-binding cassette (ABC) transporters specifically have been characterized in a variety of fungi, including *Magnaporthe oryzae* [53] and *Botrytis cinerea* [54], as important factors in evading early plant defenses and establishing infection. In the case of *B. cinerea*, the ABC transporter *BcAtrB* was found to be important in fungal tolerance to the phytoalexin camalexin [54] . *S. sclerotiorum*’s homolog of this gene (Sscle14g097690) was most induced during early infection. Further studies of this transporter proposed a likely synergistic relationship between *BcAtrB* and the detoxification enzyme *BcLCC2*, which required early activity of *BcAtrB* to reduce the accumulation of antifungal compounds and effectively degrade the target compounds in a *BcLCC2* dependent manner [84]. This suggests that in *S. sclerotiorum*-soybean interactions, ABC transporters potentially involved in toxin efflux are induced primarily early during infection (Additional file 10: Table S9), but a broader range of detoxification enzymes show either constitutive or late induction during infection (Table 3). A prime example of this is the GST *Ssbgt1* (Sscle01g003110)*,* which has been highlighted for its capacity to detoxify a range of phenolic phytoallexins from multiple plant species and was one of the most upregulated genes throughout infection (Table 3) [50]. We observed the strong induction of two other highly induced ABC transporters homologous to *Bmr1* (Sscle02g016430) and *Bmr3* (Sscle01g004040) from *B. cinerea*, which, along with *BcAtrB*, have all been shown to be induced in the presence of phytoalexins (resveratrol and/or camalexin) [55]. This regulation indicates a potentially strong phytoalexin pressure on *S. sclerotiorum* during the early stages of infection of soybean.

Another group of genes whose induction appears primarily important during early infection are those related to nitrate/nitrite (Sscle02g013580, Sscle01g005230, and Sscle02g016460) metabolism. The relationship of this nitrate assimilation pathway in pathogenicity is still largely unexplored, but the strong induction of these genes may be due to the low nitrogen availability at the early stages of infection.

Necrotrophic fungi often rely on their large repertoire of hydrolytic CWDEs and *S. sclerotiorum* is no exception [23, 85]. Over our time course analysis in soybean, *S. sclerotiorum* upregulated the expression of 178 putative CWDEs targeting a range of plant substrates (Table 2) and demonstrated distinct expression patterns for many of these enzymes. Comparative analysis across hosts revealed that among the 209 hydrolytic enzymes found to be upregulated during *B. napus* [24] infection, 55 transcripts, spanning putative arabinogalactan, callose, cellulose, lignin, lipid, mannan, protein, and starch degrading enzymes, were solely upregulated during infection of this host [24] (Additional file 17: Table S16). Conversely, 37 putative hydrolytic enzymes covering a similar range of substrates were only up-regulated during infection of soybean (Additional file 17: Table S16). This suggests that *S. sclerotiorum* deploys a specific repertoire of CWDEs depending on the host, highlighting the importance of studying this pathosystem in individual hosts.

Notable enzymes specifically deployed against soybean include a cutinase (Sscle02g013440) and polygalacturonase 2 (*Sspg2*; Sscle03g022740), both of which were highly induced in this study during early and late infection, respectively. *Sspg2* expression was only noted during early infection of sunflower [20]. In soybean, however, *Sspg2* is highly induced at all time points of infection in soybean and shows the largest differential expression in relation to culture of all PG’s at 96 HPI. The late induction of many cutinase and cutin degrading enzymes seen in this study also contradicts previous assertions that these enzymes are primarily required during the initial penetration of the host [21, 24]. Unlike many of the other CWDEs, all of the 28 identified genes coding for hemicellulose degrading enzymes were upregulated during this interaction (Table 2). Knowledge of this host-specific regulation of CWDEs is critical, both given their role as one of the most important virulence factors in necrotrophic fungal infection and in identifying the value of individual enzymes as targets in the control of SSR.

Serine proteases have been characterized as virulence determinants in a large number of phytopathogenic fungi, including *Colletotrichum coccodes* [86], *Fusarium oxysporum* [40], *Colletotrichum acutatum* [41], and *Acremonium Strictum* [87], and it has been noted that an elevated repertoire of subtilisins appears correlated with a necrotrophic lifestyle [42]. Seventeen serine proteases were identified and found to be upregulated *in planta*, and interestingly all showed the greatest induction at 24 hpi (Additional file 7: Table S6). Given that proteins involved in plant defense are a likely target of fungal proteolytic enzymes, this early induction may be due to an initial need to subvert the plants immune system which becomes less essential after the infection is established.

During infection, *B. cinerea* produces two major phytotoxic SMs, the sesquiterpene botrydial and the polyketide botcinic acid [88]. Although *S. sclerotiorum* lacks homologs of the genes to synthesize botrydial, it does possess homologs of the two *B. cinerea* genes, *Bcboa6* (Sscle15g106510) and *Bcboa9* (Sscle15g106480), that work in concert to synthesize botcinic acid [88]. Previous studies of *S. sclerotiorum* phytotoxin production, however, failed to identify botcinic acid [49], and transcriptomic analysis of *S. sclerotiorum* infection of *B. napus* only detected upregulation of Sscle15g106510 *in planta* [24]. Surprisingly, both Sscle15g106510 and Sscle15g106480 were up-regulated during infection of soybeans, with peak expression of both genes at 96 hpi. *Bcboa6* and *− 9* belong to a larger SM gene cluster, which is largely conserved within the *S. sclerotiorum* genome, and appears to be upregulated during infection (Fig. 6). Our results provide the first indication that a phytotoxic analog of botcinic acid may play a role in *S. sclerotiorum* pathogenesis in soybean. Early characterization of botcinic acid identified the metabolite as a broad spectrum phytotoxin which induced chlorosis and necrosis in plant tissue [89], but the late induction of this SM cluster during soybean infection indicates that this is unlikely to be the primary role of this metabolite. More recent studies have proposed that botcinic acid may have antifungal properties as well [90]. This suggests that the induction of the botcinic acid SM cluster at 96 hpi may serve a dual role in plant colonization, both to induce PCD to kill the host and also to inhibit competitive colonization of the host by other saprophytic fungi.

Fifty-seven putative secreted effectors were observed to be upregulated and/or differentially regulated during infection, consisting of a mixture of previously characterized effectors, such as *Ssv263* [15], *Ssssvp1* [18]*, Ssnep2* [16]*, Ss-rhs1* [91]*,* and *Ssitl* [17]*,* and others which are highly induced, but whose functions are yet to be determined (Sscle07g055350, Sscle06g050280, Sscle05g045060, Sscle06g048920, Sscle06g055310, Sscle11g080900). Although effectors are known to be secreted in “waves” corresponding to the various stages of infection, many of the most highly upregulated putative effectors were upregulated at all stages of infection in comparison to culture, indicating a more constitutive requirement during *S. sclerotiorum* infection (Table 4) [61, 62]. Despite this, some trends can still be deduced. All three of the effectors characterized as inducers of PCD (*Ssssvp1, Ssnep2, and Sscp1*) showed the greatest expression at 96 hpi (Table 4). This regulation is similar to that of the SM gene cluster putatively controlling botcinic acid biosynthesis, indicating that genes responsible for phytotoxicity and PCD induction in the host become increasingly active over the course of colonization. Alternatively, other putative effectors whose roles are more likely related to avoiding detection by the plant, such as the putative chitin binding Sscle08g068200 and Sscle07g062010, or undermining plant innate immunity, such as the chorismate mutase Sscle16g111080 [92], are more highly expressed during early infection (24–48 hpi) (Table 4). The recent discovery that the pathogen goes through a short biotrophic phase during early infiltration [93] supports a model whereby it prioritizes the subversion/avoidance of plant resistance mechanisms early in infection and increases phytotoxic activity as the infection progresses, similar to prototypical hemibiotrophs [61]. This data also suggests that other, poorly characterized effectors with similar patterns of high expression in either early (*Ssv263* [15], *acp1* [39], Sscle15g106630, etc.) or late (Sscle07g055350, Sscle05g041050, Sscle04g040080, etc.) infection may be involved in these same processes.

Oxalic acid (OA) is a key virulence factor in the pathogenic development of *S. sclerotiorum* [8, 10, 12]. It is known to upregulate ROS within the host, leading to the cell suicide and the establishment of disease [13, 14, 66]. Thus, redox regulation and OA biogenesis are important factors to consider when studying a particular *S. sclerotiorum*-host interaction. Surprisingly the oxaloacetate acetylhydrolase gene (*Ssoah1*) primarily responsible for OA biogenesis was neither upregulated in comparison to culture nor between early and late infection. A previous study which noted a similar phenomenon posited that an alternate route of OA biosynthesis may be utilized during early infection [19], but high expression of *Ssoah1* throughout infection and in culture indicates that the gene likely has an important role across all environments, thus precluding it from a differential expression analysis.

The upregulation of ROS by OA serves an important role in killing the host, but as ROS is broadly toxic to all life, *S. sclerotiorum* contains a number of ROS scavenging enzymes to maintain its own redox homeostasis during infection. In this study, four catalases (*Scat1*; Sscle04g037170, Sscle03g026200, Sscle05g047950, and Sscle15g104430), four peroxidases (Sscle03g024750, Sscle01g000730, Sscle07g058850, and Sscle06g053530), a superoxide dismutase (*Sssod1*; Sscle03g025030), and a peroxiredoxin (Sscle06g048910) were found to be upregulated in comparison to culture (Additional file 14: Table S13). These ROS scavenging enzymes showed unique temporal regulation over the course of infection, with the peroxiredoxin and two of the peroxidases (Sscle03g024750 and Sscle01g000730) being most upregulated at 24 hpi but all four of the catalases and the remaining two peroxidases demonstrating greatest expression at 96 hpi (Additional file 14: Table S13). Increased ROS scavenging activity during later infection corresponds with the likely accumulation of ROS required for the widespread cell death seen at 96 hpi. Early ROS scavenging activity may play a role in dampening the oxidative stress to the fungus as well, but the possible roles in fungal development cannot be overlooked as it’s well established that ROS plays a pivotal role in intracellular signaling and cell–cell interactions [94].

Finally, an examination of *S. sclerotiorum’s* transcriptome during infection of a resistant soybean line elucidated the reaction of the pathogen to a successful defense response by its host. During the resistant line infection, we detected increased expression of several secondary metabolism genes related to melanin biosynthesis as well as three putatively hydrophobic cell surface proteins (i.e. hydrophobins). While melanin biosynthesis is known to be induced by fungus in the presence of oxidative and/or osmotic stress [100], hydrophobins are often associated with fungal adhesion, infiltration, and suppression of host immune responses [102–105]. Previous studies on the hydrophobin-like gene, *TasHyd1*, from *Trichoderma asperellum* showed it to be vital in maintaining proper fungal wettability and in the adhesion of the fungus to the hosts roots [95]. Another function of hydrophobin-like genes was demonstrated in the human pathogen *Aspergillus fumigatus*, where it was shown that the protein served to mask the pathogen from recognition and the subsequent induction of innate immunity [96]. This function is of particular interest given that the same study demonstrated that melanin was an inducer of this immune response, providing a framework by which *S. sclerotiorum* may upregulate hydrophobins in concert with melanin to avoid triggering plant defenses. Although we cannot currently state whether the upregulation of these hydrophobic cell surface proteins is in response to chemical stress from the plant, increased difficulty in initial penetration, or an attempt to shield the pathogen from detection, it is clear that the fungus shows a distinct transcriptomic response to the resistant line at 24 hpi despite a lack of phenotypic differentiation until post-48 hpi. The regulation of these hydrophobins and the increase in melanin biosynthesis may play a pivotal role in either fungal penetration or the early suppression of innate immunity, which would be an important requirement of *S. sclerotiorum* during its early biotrophic phase of infection [93].

## Conclusions

Given the broad host range of *S. sclerotiorum* and the repertoire of virulence factors contained within its genome, transcriptomic analysis of individual *S. sclerotiorum*-host interactions is critical to future disease control efforts. In this study, gene expression of *S. sclerotiorum* was measured over the course of a compatible soybean infection. During early infection the pathogen prioritizes penetration and establishment by upregulating transporters involved in toxin efflux, nitrogen metabolism, and a variety of putative secreted effectors with likely roles in the suppression of plant immunity. As infection progresses the pathogen expresses a host-specific range of CWDEs and detoxification enzymes to release sugars from the plant cell walls and degrade antimicrobial compounds being produced as a response to colonization. By the later stages of infection, disease is well established and *S. sclerotiorum* appears to prioritize the transport of newly available carbohydrates, the induction of PCD in the host, and the formation of sclerotia as overwintering structures.

To study the response of *S. sclerotiorum* to a resistant (non-compatible) host, gene expression was compared to a susceptible interaction during early infection. Clear regulatory differences were identified in the production of melanin and hydrophobic surface proteins in the resistant interaction potentially indicating an attempt by the pathogen to either evade detection and/or defend against antifungal compounds. Knowledge of the mechanisms utilized by *S. sclerotiorum* to overcome either typical or novel mechanisms of plant resistance is invaluable in the pursuit of stable durable resistance to SSR and will be the focus of further research.

## Methods

### Plant and fungal material

For both the plant inoculations and culture controls, a wild type isolate of *S. sclerotiorum* strain 1980 was grown on potato dextrose agar (PDA) [10]. Strain 1980 was utilized as it is both a common laboratory strain of *S. sclerotiorum* and the genome sequenced strain used for downstream bioinformatic analysis in this study. Plant inoculations utilized agar plugs directly from this PDA culture, but culture controls were further cleaned and grown in glucose minimal (GMM) media prior to extraction. Fresh mycelia from PDA plates were scraped and then filtered through Miracloth (Darmstadt, Germany). Collected mycelia were washed (40 °C, 4000 rpm, 10 min) twice with sterile autoclaved water and used to inoculate 100 ml of GMM [97]. The GMM cultures were grown on a shaker for 48 h (150 rpm, 25 °C). Mycelia was collected by filtering through Miracloth (Darmstadt, Germany) and immediately frozen in liquid nitrogen prior to RNA extraction.

Two recombinants inbred lines of soybean (*Glycine max*), 91–145 and 91–44, were used in this study. Both lines were previously developed and evaluated as germplasm stock to increase quantitative resistance to SSR in soybean breeding programs, but are not commercially available [27]. Lines can be made available upon request to the corresponding author. Both the resistant 91–145 (R) and the susceptible 91–44 (S) lines were developed utilizing W04–1002 (P1), a SSR resistant parental line, and LN89–5717 (PI 5745542), a SSR-susceptible parental line demonstrating other desirable pathogen resistance traits [27]. Plants were supplemented with fertilizer (Miracle-Gro) every two weeks and maintained in the greenhouse or growth chamber at 24 ± 2 °C with 16-h light/8-h dark photoperiod cycle.

### Fungal inoculation

Four week old soybean plants were infected with *S. sclerotiorum* by petiole inoculation as previously described [66], using a 1 ml pipette tip agar plug of actively growing fungal hyphae. Plant tissue was sampled by cutting horizontally above and below (1.5 cm) the node of the inoculated petiole with a clean straight-edge razor. Tissue samples were then immediately frozen in liquid nitrogen prior to RNA extraction. The experimental design was completely randomized and consisted of three biological replicates for each of the treatments. For each of the experimental conditions, stem segments (∼3 cm, first internode) from 2 different plants were pooled together to form one of three biological replicates.

### RNA extraction and library preparation

Total RNA was extracted from soybean stem tissues and infecting *S. sclerotiorum* using a modified TRIzol™ Reagent protocol (Invitrogen Corp., Carlsbad, CA, USA). Further, samples were cleaned using the RNeasy Plant Mini Kit (Qiagen, Hilden, Germany). RNA concentration and purity was determined by Nanodrop (Thermo Fisher Scientific, Wilmington, DE) and sample quality was assessed using an Agilent Bioanalyzer 2100 and an RNA 6000 Nano Kit (Agilent Technologies, Santa Clara, CA). Three biological replicates were generated per treatment.

Library preparation was performed at the University of Wisconsin – Madison Biotechnology Centre (Madison, WI, USA). Individually indexed libraries were prepared using the TruSeq RNA Sample Preparation v2 kit according to the manufacturer’s instructions (Illumina, San Diego CA, USA). Library concentrations were quantified with the Qubit HS DNA kit (Thermo Fisher Scientific, Wilmington, DE). The size and quality of the libraries were evaluated with an Agilent Bioanalyzer 2100 and an Agilent DNA 1000 kit (Agilent Technologies, Santa Clara, CA) and the libraries were sequenced using Illumina HiSeq2500 (1X100bp) (Illumina, San Diego CA, USA).

### Quality check and sequence analysis

Illumina raw read data quality was verified with FastQC (http://www.bioinformatics.babraham.ac.uk/projects/fastqc/). The soybean and *S. sclerotiorum* genomes were collected from Phytozome v12.1 (https://phytozome.jgi.doe.gov/pz/portal.html#!bulk?org=Org_Gmax) and the Broad institute (https://www.broadinstitute.org/fungal-genome-initiative/sclerotinia-sclerotiorum-genome-project), respectively [23, 98]. Raw sequence reads were mapped to both genomes using the Subjunc aligner from Subread [99]. Alignments were compared to the gene annotation GFF files for both organisms (Soybean: Gmax_275_Wm82.a2.v1.gene.gff3 [98], *S. sclerotiorum*: sclerotinia_sclerotiorum_2_transcripts.gtf [23]) and raw counts for each gene were generated using the feature Counts tool from Subread. The raw counts data were normalized with the trimmed mean of means (TMM) normalization method using edgeR package [100]. The normalized gene counts were transformed to log_2_ scale using the voom method from the R Limma package, then used to generate differential expression (log_2_FC) values [101, 102]. DEGs (log_2_FC > 1 or < − 1) were generated for *S. sclerotiorum* genes from the comparison of inoculated soybeans at different timepoints to the culture control (FDR < 0.05), between early (24 and 48 hpi) and late (96 hpi) infection (FDR < 0.05), and between the timepoints of resistant (R) and susceptible (S) line infections (*p*-value < 0.01). The different statistical threshold applied in the R vs S comparison (p-value < 0.01) was utilized to account for a higher standard deviation in the R line replicates which precluded the usage of the more conservative FDR < 0.05. To reduce the likelihood of false positives from low transcript genes, samples were filtered to remove DEGs in which all timepoints had average RPKM values < 1. To utilize the most recent nomenclature in describing *S. sclerotiorum* genes, all gene loci were updated to the annotations assigned in the most recent full genome sequence of *S. sclerotiorum* [60] (Additional file 18: Table S17). Genes which were removed or combined in the most recent resequencing were removed from the analysis.

### Gene annotation and functional distribution characterization

Putative annotations were assigned to all genes using the Blast2Go software [103]. Select genes were also analyzed for conserved domains, characterized homologues, and closely related pathogenic fungi using BlastP [104]. Functional categorization of DEGs was done using the FunCat database (FunCatDb) and *S. sclerotiorum’s* annotated proteins (p3_r20263_Scl_scler). Functional distribution characterization was generated using the FunCat functional distribution tool, which applied a site-specific hypergeometric distribution to identify overrepresented functional categories (Bonferroni correction < 0.05) [31].

### RNA seq validation using reverse transcription quantitative PCR (RT-qPCR)

Stems were harvested and immediately frozen in liquid nitrogen. RNA was isolated using the above-mentioned protocol and then treated with RNase-free DNaseI (NEB Inc., Ipswich, MA, USA). The RNA was reverse transcribed using the AMV First-Strand cDNA Synthesis Kit (NEB Inc., Ipswitch, MA) and oligo-dT primer according to the manufacturer’s instructions. RT-qPCR was performed using a SensiFAST SYBR No ROX Kit (Bioline USA Inc., Taunton, MA, USA). Each reaction consisted of 5 μL of SensiFAST SYBR No-ROX Mix, 1 μL of 1:10-fold diluted template cDNAs, and 0.4 μL of 10 μM gene-specific forward and reverse primers in a final volume of 10 μL. Primers were either culled from the literature or designed using Primer3 software [105, 106] for the amplification of gene fragments that were approximately 100–250 bp in length and with an annealing temperature of 60 °C (Additional file 1: Table S1). The primer specificity was checked in silico against the NCBI database through the Primer-BLAST tool (http://www.ncbi.nlm.nih.gov). RT-qPCR was performed on a CFX96 real-time PCR system (Bio-Rad, Hercules, CA). The run conditions were: 2 min of initial denaturation at 95 °C; 95 °C for 5 s, 58 °C for 10 s, and 72 °C for 20 s (40 cycles). The relative expression of genes was calculated using the 2-ΔΔCt method [107] with the fungal β-tubulin gene (Sscle02g015170) used as an endogenous control. Three biological replicates quantified for each sample.

## Additional files

Additional file 1: **Table S1.** Primers used in the validation of RNA sequencing data. All primers were either designed for this study or culled from literature. (XLSX 217 kb)

Additional file 2: **Table S2.** All genes differentially expressed over the progression of S line infection. DEGs were generated through the comparison of average RPKM values between early infection (24 and 48 hpi) and late infection (96 hpi). FDR < 0.05; Average RPKM > 1; logFC ≥ 1 or ≤ -1. (XLSX 111 kb)

Additional file 3: **Table S3.** Characterization and functional distribution of genes found to be differentially regulated over the progression of S line infection. (A) LATE genes (B) EARLY genes. *P*-value, Bonferroni correction, and FDR are all given, but only samples with a Bonferroni correction < 0.05 were considered in the analysis. (XLSX 66 kb)

Additional file 4: **Figure S1.** Graph detailing select categories of genes that were upregulated either EARLY (Orange) or LATE (Blue). Genes were categorized using based on Blast2GO annotation or characterized homologues. (PDF 21 kb)

Additional file 5: **Table S4.** All genes found to be differentially expressed over the course of infection (FDR<0.05) which were similarly identified within sclerotial exudates in the Liang et al 2010 study. (XLSX 12 kb)

Additional file 6: **Table S5.** All genes which were differentially expressed between three timepoints of the S line infection and the culture control (C) (FDR<0.05; logFC>1 or logFC<-1). “Positively Regulated?” denotes genes in which at least one timepoint had a logFC>1. (XLSX 249 kb)

Additional file 7: **Table S6.** All cell wall degrading and related hydrolytic enzymes upregulated at some timepoint of the S line infection in comparison to the culture control (FDR<0.05; logFC>1). (XLSX 23 kb)

Additional file 8: **Table S7.** All putatively assigned secondary metabolite synthesis genes which were upregulated at some timepoint of the S line infection in comparison to the culture control (FDR<0.05; logFC>1). (XLSX 11 kb)

Additional file 9: : **Table S8.** All putatively assigned cytochrome P450 genes which were upregulated at some timepoint of the S line infection in comparison to the culture control (FDR<0.05; logFC>1). (XLSX 11 kb)

Additional file 10: : **Table S9.** All putatively assigned transporter proteins which were upregulated at some timepoint of the S line infection in comparison to the culture control (FDR<0.05; logFC>1). (XLSX 16 kb)

Additional file 11: **Table S10.** All putatively assigned transcription factors which were differentially regulated at some timepoint of the S line infection in comparison to the culture control (FDR<0.05; logFC>1 or logFC<-1). “Positively Regulated?” denotes genes in which at least one timepoint had a logFC>1. (XLSX 14 kb)

Additional file 12: **Table S12.** Average expression analysis of all S line timepoints. “Ave. Exp.” is calculated as the log2 of the average counts per million (CPM) for each sample. CPM is generated from RPKM values to normalize for sequencing depth among genes and replicates. (XLSX 1562 kb)

Additional file 13: **Table S11.** Raw RPKM value for S v C timepoint comparison (A) 24 hpi (B) 48 hpi (C) 96 hpi (XLSX 4311 kb)

Additional file 14 **Table S13.** All putatively assigned ROS scavenging enzymes which were differentially regulated at some timepoint of the S line infection in comparison to the culture control (FDR<0.05; logFC>1 or logFC<-1). “Positively Regulated?” denotes genes in which at least one timepoint had a logFC>1. (XLSX 12 kb)

Additional file 15: **Table S14.** Timepoint comparison of R and S lines. LogFC>1 indicates higher expression in the R line infection. LogFC<-1 indicates higher expression in the S line infection. (XLSX 36 kb)

Additional file 16: **Table S15.** Characterization and functional distribution of genes found to be differentially regulated between the R and S line infections. (A) DEGs at 24 hpi (B) DEGs at 96 hpi. P-value, Bonferroni correction, and FDR are all given, but only samples with a Bonferroni correction < 0.05 were considered in the analysis. (XLSX 59 kb)

Additional file 17: **Table S16.** Comparison of S. sclerotiorum hydrolytic enzymes upregulated during infection of either soybean (This study) or *B. napus* [24]. (XLSX 36 kb)

Additional file 18: **Table S17.** Key describing the relationship between version one [23] and version two [60] gene loci annotations within the *S. sclerotiorum* genome. (XLSX 212 kb)

## Abbreviations

ABC: ATP binding cassette
Ave. Exp: Average Expression BlastP: Protein BLAST
C: Culture control
CFEM: Common fungal extracellular membrane
CWD: Cell wall degrading
CYP450: Cytochrome P450
DBD: DNA-binding domain
DEG: Differentially expressed gene
EARLY: Genes showing up-regulation early in infection
FDR: False discovery rate
GMM: Glucose minimal media
GST: Glutathione S-transferase
HPI: Hours post inoculation
LATE: Genes showing up-regulation late in infection
MFS: Major facilitator superfamily
NADPH: oxidase
NOX: logFC: Log_2_ fold change
NRPS: Non-ribosomal peptide synthase
OA: Oxalic acid
PCD: Programmed cell death
PDA: Potato dextrose agar
PG: Polygalacturonase
PKS: Polyketide synthase
PTI: Pattern triggered immunity
R: Resistant line
RIL: Recombinant inbred lines
ROS: Reactive oxygen species
RPKM: Reads Per Kilobase of transcript, per Million mapped reads
S: Susceptible line
SM: Secondary metabolite
SOD: Superoxide dismutase
SSR: Sclerotinia stem rot
TF: Transcription factor
TM: Transmembrane
TMM: Trimmed mean of means
