# Supplementary figures and images for: Gene regulation of Sclerotinia sclerotiorum during infection of Glycine max: on the road to pathogenesis
Source: BMC Genomics. 2019 Feb 26;20:157. doi: 10.1186/s12864-019-5517-4 (PMC6390599; doi:10.1186/s12864-019-5517-4)

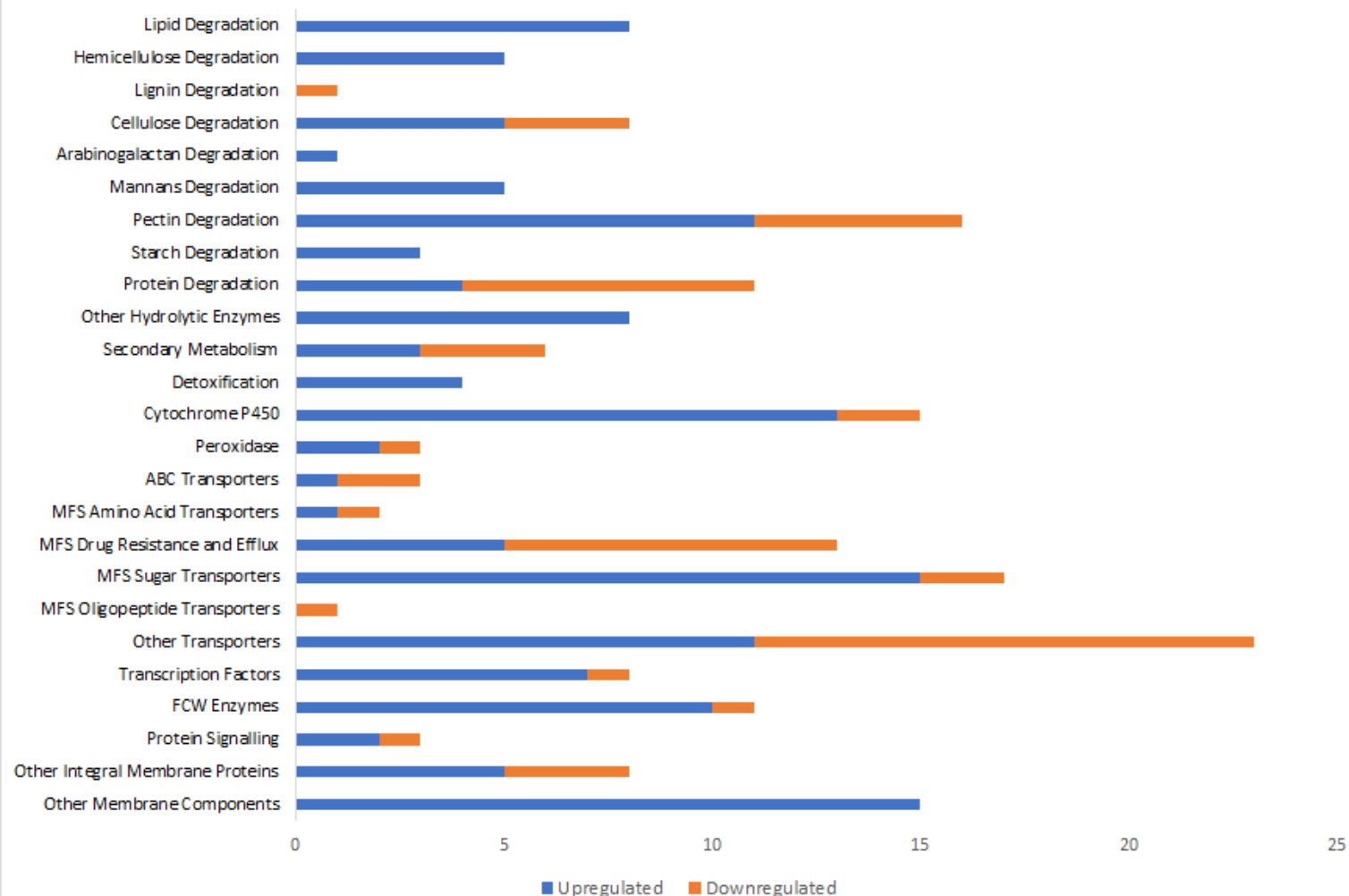

Supplement: Supplementary file 4 — Figure S1. Graph detailing select categories of genes that were upregulated either EARLY (Orange) or LATE (Blue). Genes were categorized using based on Blast2GO annotation or characterized homologues. (PDF 21 kb) [file 12864_2019_5517_MOESM4_ESM.pdf]
